# Red blood cell distribution width as a prognostic biomarker for viral infections: prospects and challenges

**DOI:** 10.2217/bmm-2021-0364

**Published:** 2021-11-17

**Authors:** Oloche Owoicho, Kesego Tapela, Charles O Olwal, Alexandra L Djomkam Zune, Nora N Nganyewo, Osbourne Quaye

**Affiliations:** ^1^Department of Biochemistry, Cell & Molecular Biology, West African Centre for Cell Biology of Infectious Pathogens (WACCBIP), College of Basic & Applied Sciences, University of Ghana, Accra, Ghana; ^2^Department of Biological Sciences, Benue State University, Makurdi, Nigeria; ^3^West African Network of Infectious Diseases ACEs (WANIDA), French National Research Institute for Sustainable Development, Marseille, France; ^4^Medical Research Council Unit, The Gambia, at London School of Hygiene & Tropical Medicine, Banjul, The Gambia

**Keywords:** anisocytosis, prognostic biomarkers, red blood cell distribution width, red blood cells

## Abstract

Viral diseases remain a significant global health threat, and therefore prioritization of limited healthcare resources is required to effectively manage dangerous viral disease outbreaks. In a pandemic of a newly emerged virus that is yet to be well understood, a noninvasive host-derived prognostic biomarker is invaluable for risk prediction. Red blood cell distribution width (RDW), an index of red blood cell size disorder (anisocytosis), is a potential predictive biomarker for severity of many diseases. In view of the need to prioritize resources during response to outbreaks, this review highlights the prospects and challenges of RDW as a prognostic biomarker for viral infections, with a focus on hepatitis and COVID-19, and provides an outlook to improve the prognostic performance of RDW for risk prediction in viral diseases.

Infectious diseases, caused by bacteria, viruses, parasites and fungi, remain a global health concern. In recent history, viruses have emerged as a major global threat. Humanity has been plagued by myriads of viral infections, including Zika virus, Ebola virus [1] and the current SARS-CoV-2 pandemic. Viral threats are partly attributable to the ability of the viruses to mutate frequently and adapt to different hosts [2]. To overcome the threat of viral pandemics, humanity must be able to effectively identify emerging lethal viruses and predict the prognosis of the viral disease. Especially where there are limited resources, identification of individuals at risk of mortality or severe morbidity is required for prioritization of resources and effective management of a new viral disease outbreak, such as the COVID-19 pandemic. To this end, a constant search for biomarkers that can predict prognosis of many diseases, including those caused by viruses, is important.

Red blood cells (RBCs) are the major cellular component of circulating blood, reaching approximately 5 million cells per cubic ml of blood. With a life span of about 100–120 days, erythrocyte production and senescence are maintained in constant equilibrium. Normally, a red cell has a disc-like form; a well-hemoglobinized cytoplasmic rim with a central pallor covering the inner third of the cell. Deviations in RBC morphology (size, shape, color, contents/inclusion or distribution) is likely a diagnostic marker of disease entities [3]. Typically, there are different qualitative and quantitative measures of RBCs; these include mass, volume, count, hematocrit and hemoglobin concentration. These measurements are expected to be within certain limits for an age bracket and sex in a specified population [3].

Red blood cell distribution width (RDW) is an inexpensive and readily available laboratory parameter that is reported on the complete blood count with many modern hematology analyzers. RDW measure reflects the extent of anisocytosis, a condition that is characterized by pronounced heterogeneity in the volume of circulating erythrocytes [4], and may act as a prognostic factor in several diseases [5]. RDW quantifies the variation of individual RBC volumes, which vary from one cell to the other, and for the same cell as it circulates during its lifespan [6,7]. Elevated RDW is associated with an increased risk for the morbidity or mortality of a plethora of noncommunicable and communicable diseases or conditions [8–10].

This review provides an overview of anisocytosis and the prospects and challenges of RDW as a predictor of morbidity and mortality that could be associated with viral diseases.

## Anisocytosis

RBCs are formed in the bone marrow from erythroid progenitors through a process of erythropoiesis. The process involves the production of proerythroblasts, which develops to erythroblasts and reticulocytes, and finally mature into erythrocytes. The features of a mature RBC is a disc-like element with a diameter of 6–8 μm and a mean corpuscular volume of 80–100 femtolite (fL). However, RBC can have abnormal volumes, which can either be reduced or increased, conventionally called microcytic or macrocytic RBCs, respectively [11]. This is caused by the disruption of erythropoiesis due to derangement of many biological conditions/processes such as aging, inflammation, oxidative stress, nutritional deficiencies and impaired renal function [12]. Microcytosis is uncommon, and is featured by RBCs with a mean corpuscular volume of less than 80 fL, as seen in microcytic anemia [13]. Macrocytosis is generally defined as the presence of large-sized erythrocytes in peripheral venous blood [14].

Anisocytosis is an RBC size disorder which occurs during the cell division processes. A study of normal chicken RBC precursors showed that a drastic reduction of G1 and downregulation of D-cyclins and cyclin-dependent kinase (CDK) 4 genes alter RBC size control [15] . The D-cyclins regulate cell cycle by activating the CDK4 and 6 proteins, which phosphorylate retinoblastoma tumor suppressors to release transcription factors that promote the G1-S phase transition of the cell cycle [16]. An increased RBC size and decreased RBC count have been observed in mice without *CCND3*, a gene-encoding cyclin D3 [17]. Both human and mouse primary erythroid cells also demonstrated that cyclin D3 controls RBC size and count by regulating the number of cell divisions that RBC progenitors undergo during their terminal differentiation [18]. The downregulation of cyclin D3 regulates erythroid proliferation through its interaction with CDK4 and CDK6, and in turn, causes a downregulation of Pu.1, an antagonist of GATA-1 function [11]. These changes in gene expression alter cell cycle progression, causing the loss or alteration of cell size control, resulting in fewer and larger-sized RBCs.

A physiological size heterogeneity of RBC (microcytosis or macrocytosis) in adult human blood is usually measured in terms of RDW [11]. The RDW may be influenced by physiological (e.g., pregnancy, aging or physical exercise) or pathological (e.g., iron deficiency anemia, inflammation and oxidative stress) factors [12]. Persistent inflammation, which can be due to infections, is associated with higher RDW [19], and studies have shown that proinflammatory cytokines inhibit erythrocyte maturation, causing an increase in immature erythrocytes circulating in blood, and resulting in higher RDW values [19]. Decrease in antioxidants results in oxidative stress, which potentially increases RDW by decreasing the rate of erythroid maturation and lifespan [20]. For instance, selenium and carotenoids have been shown to protect erythrocytes from increased RDW by preventing it from oxidative damage [21].

High RDW has been suggested as a marker for severity of viral disease. However, the precise mechanism by which the viral infections induce high RDW remains unclear. Viral infection possibly activates NF-κB and other immune factors which then initiate inflammation [22], and advances to chronic inflammation. Viral-induced chronic inflammation impairs erythrocyte maturation and leads to changes in erythropoiesis or underproduction of the hormone erythropoietin [23], and therefore likely to account for the positive correlation between RDW levels and severity of viral infections. More detailed studies are however needed to decipher the molecular mechanisms that underlie the prognostic value of RDW in viral diseases.

## RDW as a prognostic biomarker

The possible use of RDW as a prognostic biomarker for severe morbidity and/or mortality that are caused by various malignancies have been highlighted, and these include renal cell carcinoma [24], multiple myeloma [25,26], lung cancer [27], gastric cancer [28], ovarian cancer [29], breast cancer [30] and hematological malignancies [31]. RDW has also been shown to be a novel predictive biomarker for cardiovascular diseases [32,33], progression to end-stage renal disease among diabetic patients [34], and a significant diagnostic and prognostic marker for pre-eclampsia [35]. Collectively, these studies highlight an emerging utility of RDW as a diagnostic or prognostic indicator of a wide range of noncommunicable diseases.

RDW has shown significant potentials for differential diagnosis, morbidity, and mortality predictions of infectious diseases, including parasitic [36] and bacterial diseases [37], community-acquired pneumonia [38], infective endocarditis [39], sepsis due to Gram-negative bacteria [40] and viral diseases [41,42]. The parameter is increasingly gaining attention as a possible, easily available and cost-effective biomarker for diagnosis and prognosis of infectious diseases. Interestingly, in a study which evaluated 3883 virus- or/and bacteria-infected patients, RDW in combination with hemoglobin level was observed to significantly differentiate viral from bacterial infections [43].

Studies that have explored RDW as a diagnostic or prognostic biomarker of viral diseases have mostly focused on hepatitis B virus-associated liver disease and COVID-19 (Table 1). However, the parameter has also been explored for other viral diseases such as HIV/AIDS [44] and Epstein–Barr virus infectious mononucleosis [45]. Subsequently, we discuss a wide-range of studies on the potentials of RDW as a prognostic biomarker for hepatitis virus-associated diseases and COVID-19.

**Table 1.** Highlights of studies on red blood cell distribution width for prognosis of viral diseases and their major findings.

Viral disease	Author (year)	Study design	Main findings	Ref.
COVID-19	Bergamaschi *et al.* (2021)	Observational study of 206 COVID-19 confirmed patients	RDW elevation significantly associates with COVID-19-associated mortality	[43]
COVID-19	Wang *et al.* (2020)	Retrospective cohort involving 45 COVID-19 patients (age: 16–62 years)	A combination of NLR and RDW-SD differentiates moderate from severe COVID-19 case with 90% sensitivity and 84.7% specificity. NLR and RDW-CV, combined, also differentiates the two categories with 90% sensitivity but with 82.4% specificity	[44]
COVID-19	Lanini *et al.* (2020)	Longitudinal cohort study of 379 COVID-19 patients (61.67 ± 15.60).	RDW is elevated in patients who died of COVID-19	[45]
COVID-19	Karampitsakos *et al.* (2020)	Observational study of 193 hospitalized COVID-19 patients (mean age: 61 years)	Baseline RDW ≥14.5% is strongly associated with an increased risk of mortality	[46]
COVID-19	Mertoglu *et al.* (2020)	Retrospective study of 555 COVID-19 patients	RDW is elevated in a significant number (36.8%) of the patients	[50]
COVID-19	Lin *et al.* (2020)	Retrospective study of 68 COVID-19 patients (mean age: 53.6 ± 11.4)	There is no significant difference in RDW between patients with mild and severe COVID-19 disease	[47]
COVID-19	Lorente *et al.* (2020)	Prospective multicenter study of confirmed COVID-19 patients admitted to an ICU	Elevated RDW is associated with 30-day mortality in COVID-19 patients admitted to ICU. The RDW cutoff of 13 has 88% sensitivity and 45% specificity in predicting COVID-19 mortality in ICU patients	[48]
COVID-19	Hornick *et al.* (2020)	Longitudinal cohort study (age: 53–75 years)	There is a continued relationship between RDW and mortality; 1% increase in RDW and a 10-year increase in age confer a similar mortality effect. RDW cutoff of 13.6% predicts 30-day mortality with 80% sensitivity and 59% specificity while a cutoff of 14.5% has 72 and 81% sensitivity and specificity, respectively	[49]
COVID-19	Asan *et al.* (2021)	Retrospective single-center study of 695 confirmed COVID-19 patients	RDW is not a significant positive predictor of ICU requirement in COVID-19 patients	[50]
COVID-19	Foy *et al.* (2020)	Cohort study of 1641 hospitalized adult COVID-19 patients	Increased RDW (>14.5%) at admission and increasing RDW during hospitalization are significantly associated with increase in mortality risk in COVID-19 patients	[51]
COVID-19	Liu *et al.* (2020)	Cross-sectional study of 134 type diabetics with COVID-19	RDW is not significantly associated with SARS-CoV-2 infection severity	[52]
COVID-19	Henry *et al.* (2020)	Prospective observational study of 49 COVID-19 patients (age: ≥18 years)	RDW increase is associated with ninefold and 16-fold increase odds of SARS-CoV-2 infection severity and acute kidney failure, respectively. A 14.5% RDW cut-off value predicts SARS-CoV-2 infection severity with 81 and 64% sensitivity and specificity, respectively	[53]
COVID-19	Sharma *et al.* (2020)	Retrospective observational study of 70 COVID-19 patients	RDW is slightly elevated in symptomatic and asymptomatic SARS-CoV-2 infection	[54]
COVID-19	Pan *et al.* (2020)	Retrospective study involving 84 COVID-19 patients and 221 patients with CAP	RDW discriminates between COVID-19 and CAP fairly	[55]
COVID-19	Gong *et al.* (2020)	Retrospective study of 372 with nonsevere COVID-19 on admission	RDW is a prognostic predictor of severe COVID-19	[56]
Infectious mononucleosis	Han *et al.* (2020)	Prospective case–control, involving children with IM	RDW positively correlates with ALT, AST and GGT elevation. An RWD cutoff of 12.55% is 80.9% sensitive and 78.8% specific for indirect liver damage prediction in children with IM	[42]
Hepatitis B viral disease	Qiang *et al.* (2020)	Retrospective cohort involving 577 patients (mean age: 47 ± 11.40 years) with HBV-related ACLF	An RDW could be used as a predictor of 90-day mortality in HBV patients with ACLF; when used together with other systemic inflammatory markers (NLR, TBIL, INR and Cr), the RDW predictive power is higher than MELD scores	[57]
Hepatitis B viral disease	Li *et al.* (2020)	Retrospective cohort involving 174 patients (mean age: 53.6 ± 11.40 years) diagnosed with HBV-DeCi	RDW increase associates significantly with mortality in HBV-DeCi patients but is not an independent predictor of 28-day mortality	[58]
Hepatitis B viral disease	Wang *et al.* (2020)	Prospective case–control, involving 1967 CHBD and 325 healthy controls	RDW positively correlates with CHB severity and is an independent predictor of liver-related mortality in CHBD patients	[44]
Hepatitis B viral disease	Zhu *et al.* (2019)	Retrospective case–control study involving 752 CHBD and 160 healthy controls	RDW could differentiate CHB-related cirrhosis from active CHB and inactive CHB carriers	[59]
Hepatitis E viral disease	Wu *et al.* (2019)	Case–control study involving 262 HEV infected patients	RDW to lymphocyte ratio is a predictor of liver failure in HEV-infected persons	[60]
Hepatitis B viral disease	Qin *et al.* (2018)	Retrospective cohort study of 245 patients with HBV-ACLF	RDW is an independent predictor of 90-day mortality in patients with HBV-ACLF. MELD score and RDW predict a short-term prognosis of HBV-ACLF better than MELD score	[61]
Hepatitis B viral disease	Cai *et al.* (2018)	Cohort study involving 345 patients with various manifestations of CHBD	Although RDW is significantly higher in patients with liver failure compared with LC, CHB and HS, it is not an independent mortality predictor in patients with HBV-ACLF	[62]
HIV/AIDS	Zhang *et al.* (2018)	Cross-sectional study involving 158 HIV-infected patients on ART (median age: 50 years)	RDW correlates with systemic inflammatory biomarkers and T-cell dysregulation in HIV-infected persons on stable ART	[41]
Hepatitis B viral disease	Ferdous *et al.* (2018)	A cross-sectional study involving 40 patients with CHBD	RDW to platelet ratio correlates positively and significantly with hepatic fibrosis staging in patients with CHBD	[63]
Hepatitis B viral disease	Zhang *et al.* (2017)	Retrospective study involving 172 patients with HBV-DeCi	RDW is an independent predictor of HBV-DeCi severity, with a sensitivity of 90.7% and specificity of 100.0% at >14% cut-off value	[64]
Hepatitis B viral disease	Jin *et al.* (2017)	Retrospective study involving 122 patients with CHBD	RDW cut-off value of 17% is an independent predictor of mortality in HBV-ACLF patients. Patients with >17% RDW have lower survival rate than those with ≤17% RDW	[65]
Hepatitis B viral disease	Kai *et al.* (2016)	Case–control study involving 1462 patients with CHBD	RDW is an independent predictor of 90-day mortality in patients with HBV-DeCi, with a sensitivity of 92.16 and 66.49% specificity at 17.5% cut-off value	[66]
Hepatitis C viral disease	He *et al.* (2016)	Case–control study involving 94 patients (mean age: 47.23 ± 12.78) with HCVD	RDW and RDW to platelet ratio are predictors of cirrhosis due to HCV infection and are potential biomarkers for HCV infection severity	[70]
Hepatitis B viral disease	Gao *et al.* (2014)	Prospective (cross-sectional) study of 373 patients CHBD	In CHBD patients with ALT≥ double upper limit of healthy persons, RDW increases with increase in the viral load	[67]
Hepatitis B viral disease	Karagoz *et al.* (2014)	Retrospective case–control study involving 229 treatment-naive CHB patients (mean age: 30.9 years)	RDW is significantly elevated in CHB patients and can serve as an independent predictor of liver fibrosis	[68]
Hepatitis B viral disease	Huang *et al.* (2014)	Case–control study involving 130 patients with CHBD	RDW is increased in patients with CHB and positively correlate with the severity of HBV-related hepatic cirrhosis	[69]
Hepatitis B viral disease	Lou *et al.* (2012)	Case–control study involving 123 patients with HBV infection	RDW is significantly elevated in patients infected with HBV, associates with HBVD severity and is an independent predictor of 3-month mortality rate in HBVD patients	[70]

ALT: Alanine transaminase; ART: Antiretroviral therapy; CAP: Community-acquired pneumonia; CHBD: Chronic Hepatitis B disease; Cr: Creatinine; CV: Corpuscular volume; HBV: Hepatitis B virus; HBV-ACLF: HBV-related acute-on-chronic liver failure; HBV-DeCi: HBV-related decompensated cirrhosis; HS: Healthy subjects; ICU: Intensive care unit; IM: Infectious mononucleosis; INR: International normalized ratio of prothrombin time; LC: Cirrhosis patients; MELD: Model for end-stage liver disease; NLR: Neutrophil/lymphocyte ratio; RDW: Red blood cell distribution width; SD: Standard deviation; TBIL: Total bilirubin.

## RDW as a predictor of hepatitis virus diseases morbidity & mortality

As shown on Table 1, several studies have reported a significant elevated RDW value in patients with chronic hepatitis B infection. In addition, RDW has been considered a predictor of liver disease severity in such patients, either as an independent predictive biomarker or in combination with other hematological indices [60 69–71]. Increase in RDW correlates with viral load in chronic hepatitis B disease patients with a 100% increase in alanine transaminase above the upper limit of the enzyme in nondiseased control group [67]. In a study involving 752 patients with chronic hepatitis B and 160 healthy control, Zhu *et al.* observed that RDW could differentiate chronic hepatitis B-related cirrhosis from active and inactive chronic hepatitis B infections [59].

RDW has been shown to independently predict the severity of hepatitis B virus-related decompensated cirrhosis (HBV-DeCi) with about 91% sensitivity and 100% specificity at a cut-off value of 14% [64]. A cut-off value of 17.5% of the biomarker could predict 90-day mortality in HBV-DeCi patients with a sensitivity of 92.16 and 66.49% specificity [66], and patients with HBV-related acute-on-chronic liver failure (HBV-ACLF) with >17% RDW have lesser survival rate than those with RDW of ≤17% [65]. When used in combination with other systemic inflammatory markers such as neutrophil-lymphocyte ratio, total bilirubin, international normalized ratio and creatinine, the predictive power of the RDW-related model for 90-day mortality in HBV-ACLF patients is higher than the model for end-stage liver disease (MELD) score [57]. The MELD score is a validated predictor of survival in patients with hepatological disorders such as hepatitis, cirrhosis and acute liver failure, and it is accounted for by serum bilirubin and creatinine levels, INR and etiology of liver disease [71]. A combination of RDW and MELD score predicts a short-term prognosis of HBV-ACLF better than a MELD score alone [61].

Besides HBV-related diseases, the predictive value of RDW for other hepatitis virus diseases has been accessed. RDW, as an independent factor, and RDW-platelet ratio, have been reported as predictors of hepatitis C virus (HCV) infection severity and progression to cirrhosis in a case–control study [67]. Similarly, another case–control study observed that RDW–lymphocyte ratio predicted liver failure due to hepatitis E virus [60].

## RDW as a predictor of COVID-19 morbidity & mortality

Several studies have reported RDW elevation in severe COVID-19 patients [43,44,51,56]. Recent studies that used confirmed COVID-19 patients observed that, among the routine complete blood count parameters, only RDW was significantly associated with mortality and had predictive significance as an independent risk factor [43], and a baseline RDW ≥14.5% is strongly correlated with increased risk of mortality [72]. In a multicenter prospective observational study of COVID-19 patients that were admitted to intensive care units of six hospitals in Spain, RDW significantly associated with, and predicted the 30-day mortality after controlling for relevant confounding factors [48]. Additionally, an RDW cut-off value of 13.5% predicted a 30-day COVID-19 mortality with 80% and 59% sensitivity and specificity, respectively, whereas a cut-off of 14.5% has 72% sensitivity and 81% specificity [49]. Among COVID-19 patients admitted to ICUs, an RDW cut-off of 13% has 88% sensitivity and 45% specificity in predicting COVID-19 mortality [48], and another study found that a 14.5% cut-off value predicted disease severity with 81 and 64% sensitivity and specificity, respectively [53]. The sensitivity and specificity of RDW in predicting SARS-CoV-2 infection severity and mortality seem to differ between studies. The differences may be due to variances in clinical and/or demographic characteristics of the patients, and study and/or assay design, and therefore highlight the need for the standardization of existing protocols for evaluation of RDW as a COVID-19 prognostic biomarker. Diagnostically, RDW could also differentiate between COVID-19 and other types of community-acquired pneumonia [55], and there was no significant difference between RDW and sequential organ failure assessment as predictors of COVID-19 mortality [48].

## Limitations of RDW as prognostic biomarker for viral diseases

The limitations of RDW as a prognostic biomarker for viral diseases can be divided into technical and biological.

## Technical limitations

A major challenge of using RDW as a prognostic biomarker in diseases is the establishment of a universal RDW reference range [73], which has been difficult due to variations in RBC size measurement methods, instrumentation, standards and statistical approaches across different laboratories [65]. Commercial hematological analyzers have varying size limits and relative heights of RBC histogram employed in the calculation of RDW [57], and modern analyzers estimation of RDW is based on impedance or optical techniques, adding to the complications of varied instrumentation [66]. Moreover, there is no global agreement as to whether RDW should be expressed as standard deviation or as the coefficient of variation of erythrocyte volumes [57]. While the RDW reference range typically spans 12–15%, normal values generated largely depend on the instrumentation and population [71].

In addition to the varied instrumentation, RDW value may also be affected by pre-analytical factors such as the times and conditions of sample collection, including eating or drinking and blood transfusion prior to sample collection [43,44]. It has also been observed that if ethylenediaminetetraacetic acid is used as an anticoagulant for sample collection instead of citrate, the RDW values are falsely elevated, leading to unreliable results [61].

## Biological limitations

Several factors such as anemia, malnutrition, bone marrow depression, erythropoietin use, thyroid dysfunction, iron or vitamin B12 deficiency, cardiovascular disease, increased angiotensin II, renal or liver dysfunction, acute or chronic inflammation, among others, have been shown to affect RDW values [44,45,74 75–77. These factors, being common to many diseases, limits the potential of RDW as a biomarker [54,75–78]. The multiple factors above reduce RDW specificity as a predictive biomarker for disease progression. RDW also increases with age [45], and hence, age is a confounding factor in the use of the biomarker for prognosis prediction of viral diseases. Given the large number of factors associated with increased RDW, it may be challenging to adjust for all the possible confounding factors in a single study. Moreover, RDW is dynamic in the course of many infectious diseases, including viral diseases, hence, utilization of RDW measurement as a prognosis predictor, may not consider the dynamic changes at different stages of disease progression [47], potentially reducing its accuracy. Abnormal pathological mechanisms that usually take place in blood circulation also limit the specificity of RDW as a marker of disease prognosis [50].

## Surmounting the limitations of RDW as a prognostic biomarker for diseases

Irrespective of the limitations of RDW, it is still a promising prognostic marker for viral infections. To mitigate the limitations of RDW as a prognostic biomarker for viral diseases, the following may be considered.

First, the International Council for Standardization in Hematology should promote the standardization of RBC distribution curve analysis, which has been mostly overlooked by manufacturers in the development of instrumentation for RDW, and the choice of anticoagulant and standard deviation- or coefficient variation-based calculations. Second, since RDW varies between populations and different age groups, it will be invaluable for global geographical regions and subregions to establish age and population-specific reference ranges; this will limit errors made as a result of age or different population dynamics. Third, a prognostic model which incorporates different factors that can influence RDW and evaluate the contribution of each of these factors will be a step in overcoming the biological limitations of RDW as a prognostic biomarker for viral diseases. Otherwise, age and other confounding factors should be adjusted for in multivariate analysis when using RDW as a predictive biomarker for disease progression or mortality. Finally, since times and conditions of sample collection affect RDW levels, guidelines on proper timing and conditions for sample collection should be put in place to enable laboratories to collect the right samples at the appropriate time and under the right condition(s).

## Conclusion

The emergence of viruses is a major global threat, and early diagnosis and management of most emerging viruses is still a challenge. Hence, identification of a readily available and cost-effective prognostic biomarker will be imperative for resource allocation, reduction of morbidity and mortality, especially in cases of emerging viral diseases such as SARS-CoV-2. Compared with other biomarkers, RDW measurement is cost-effective and can be estimated without invasive techniques such as biopsy. Thus, the potentials of RDW require further research to explore its use as a good prognostic biomarker for emerging viruses, as well as to devise strategies to mitigate the limitations of using this easily obtainable predictive parameter.

## Future perspective

As demonstrated by the COVID-19 pandemic, emerging viral pathogens are ticking time-bombs, and therefore it is necessary to prepare to combat them effectively. Considering the economic burden of managing patients with viral diseases such as COVID-19, there is the need for early prediction of those likely to develop severe conditions, so that limited resources can be prioritized toward saving the lives of those who are at risk of developing severe complications. Upon overcoming the pitfalls of using RDW, as suggested in this article, RDW will likely revolutionize the prediction of viral disease prognosis and lead to appropriate and timely medical interventions. We anticipate that this review will instigate research studies that are aimed at fully understanding the interplay between RDW and many viral infections. As more detailed research reports emerge on the precise mechanisms by which viral infections influence RDW, we foresee the development of affordable, easy to use, and rapid RDW-based kits to predict patients who are at risk of developing severe viral diseases complications.

**Executive summary**

- Effective identification of emerging lethal viruses, as well as accurate prediction of disease prognosis, is imperative.
- Red blood cell distribution width (RDW) is an emerging prognostic indicator of a wide range of diseases, including viral infections.
- RDW predicts the severity and progression of hepatitis viruses and COVID-19 diseases with high sensitivities and specificities.
- RDW is an inexpensive and a readily available laboratory parameter and therefore highlights a likely enormous benefit in resource-strained countries.
- The universal use of RDW for predicting the prognosis of viral diseases currently has some limitations.
- Further research geared toward surmounting the current limitations is needed to ensure the uniform application of RDW as a prognostic biomarker for emerging viral diseases.
